# Fusion of locomotor maneuvers, and improving sensory capabilities, give rise to the flexible homing strikes of juvenile zebrafish

**DOI:** 10.3389/fncir.2013.00108

**Published:** 2013-06-07

**Authors:** Rebecca E. Westphal, Donald M. O'Malley

**Affiliations:** ^1^Department of Natural Sciences, North Shore Community CollegeLynn, MA, USA; ^2^Department of Biology, Northeastern UniversityBoston, MA, USA

**Keywords:** zebrafish, prey capture, ontogeny, lateral line, motor learning, evolution, imaging, vision

## Abstract

At 5 days post-fertilization and 4 mm in length, zebrafish larvae are successful predators of mobile prey items. The tracking and capture of 200 μm long *Paramecia* requires efficient sensorimotor transformations and precise neural controls that activate axial musculature for orientation and propulsion, while coordinating jaw muscle activity to engulf them. Using high-speed imaging, we report striking changes across ontogeny in the kinematics, structure and efficacy of zebrafish feeding episodes. Most notably, the discrete tracking maneuvers used by larval fish (turns, forward swims) become fused with prey capture swims to form the continuous, fluid homing strikes of juvenile and adult zebrafish. Across this same developmental time frame, the duration of feeding episodes become much shorter, with strikes occurring at broader angles and from much greater distances than seen with larval zebrafish. Moreover, juveniles use a surprisingly diverse array of motor patterns that constitute a flexible predatory strategy. This enhances the ability of zebrafish to capture more mobile prey items such as *Artemia*. Visually-guided tracking is complemented by the mechanosensory lateral line system. Neomycin ablation of lateral line hair cells reduced the accuracy of strikes and overall feeding rates, especially when neomycin-treated larvae and juveniles were placed in the dark. Darkness by itself reduced the distance from which strikes were launched, as visualized by infrared imaging. Rapid growth and changing morphology, including ossification of skeletal elements and differentiation of control musculature, present challenges for sustaining and enhancing predatory capabilities. The concurrent expansion of the cerebellum and subpallium (an ancestral basal ganglia) may contribute to the emergence of juvenile homing strikes, whose ontogeny possibly mirrors a phylogenetic expansion of motor capabilities.

## Introduction

Vertebrate animals begin life with only rudimentary motor skills, but these skills progress enormously over ontogenetic time. Predation is a particularly valuable skill and many predatory mammals have an extensive epoch of “play” where such skills are learned and honed. Fishes, however, must generally fend for themselves and so must continuously and effectively perform this behavior while undergoing rapid growth and changing ecological circumstances. Zebrafish commence exogenous feeding at about 5 days post fertilization (dpf). At this early developmental stage, zebrafish have an estimated 300 descending neurons that project from the brainstem to the spinal cord (Kimmel, 1982; Kimmel et al., 1982, 1985; Metcalfe et al., 1986; Gahtan et al., 2002; summarized in O'Malley et al., 2003). Over the course of about 90 days, zebrafish progress through the early larval, late larval and juvenile ontogenetic stages before reaching adulthood (Parichy et al., 2009). Effective prey capture is ongoing throughout this time, but how this capability is maintained and advanced is not known.

**Figure 1:**
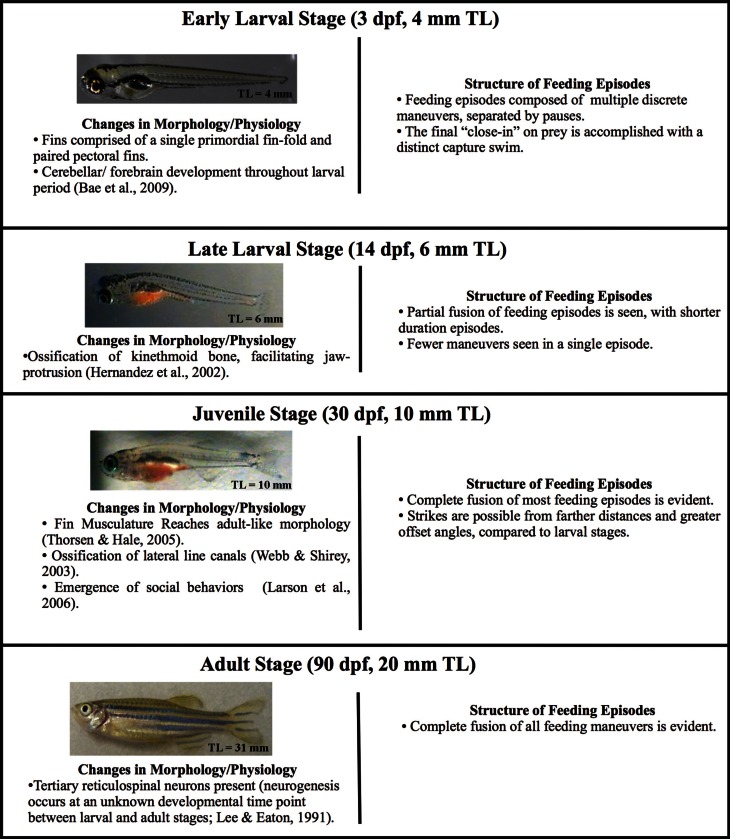
**Changes in zebrafish morphology and predatory behavior across ontogeny**. Four developmental stages (early larval, late larval, juvenile, and adult) are defined based upon total length, which defines the start of each stage. Each stage is accompanied by a representative image of a fish and the typical age at which it begins. Zebrafish develop gradually, i.e., without a distinct metamorphosis, but major changes in body form occur throughout these stages, including changes to fins, bones and body form. Morphological change is accompanied by behavioral changes, which are summarized. The early larval micrograph is courtesy of Dr. Edward Devlin.

Many physiological, morphological and behavioral changes occur throughout zebrafish ontogenesis (see e.g., Nüsslein-Volhard and Dahm, 2002; Webb and Shirey, 2003; Ghysen and Dambly-Chaudière, 2004; Hernández et al., 2005; Larson et al., 2006; Bae et al., 2009; Valente et al., 2012), with especially substantial growth from the age of first predation (about 5 dpf) to the late juvenile stage (summarized in Figure 1). One of the most conspicuous morphological developments is differentiation of the fins. Larval fish hatch with a single primordial fin fold which is eventually replaced by the caudal, dorsal, and anal fins (Nüsslein-Volhard and Dahm, 2002; McHenry and Lauder, 2006; Danos and Lauder, 2007). Concurrently, the paired pectoral fins develop from a simple endoskeletal disc to a more complex fin, including twelve actinotrichia (fin rays) in each of the bilateral pectoral fins. This is accompanied by the differentiation of pectoral fin-associated muscles and neurons (Thorsen and Hale, 2005, 2007; Green and Hale, 2012). In addition to these changes in the appendicular skeleton and associated musculature, the jaw is also gaining new function: premaxillary protrusion is facilitated by the ossification of the kinethmoid bone during late larval developmental (Hernández, 2000; Hernández et al., 2002; Staab and Hernández, 2010). Moreover, the body form is becoming more streamlined (McHenry and Lauder, 2006).

### Kinematic details

While few data are available for later life stages, the kinematics of early-larval zebrafish preying upon *Paramecium multinucleatum* have been described in some detail. Predation on these small, 200–300 μm long prey items was described using high-speed imaging. Prey tracking, at this stage, consists of a variable number and combination of orienting turns and forward swims that serve to better align the fish to its prey by decreasing its angular deviation and distance (McElligott and O'Malley, 2005; McClenahan et al., 2012; Patterson et al., 2013). The orienting turns are kinematically distinct from previously described larval turns (routine and escape) and were called “J-turns” because of their rhythmic J-bends that are unilateral, far caudal (85.5% body length) and of high amplitude (bend angles >90°). The forward swim bouts observed during tracking are indistinguishable from previously described slow swims (Budick and O'Malley, 2000). These slow swims involve rhythmic tail beats that propel the fish forward, closer to the prey. In addition, there are a variety of fin movements associated with swimming and predatory behaviors (Thorsen et al., 2004; Danos and Lauder, 2007; McClenahan et al., 2012).

The consummatory phase of larval feeding, termed a capture swim, consists of a distinctive maneuver in which “fine axial motor control” generates rapid, precise acceleration through the prey item's location (Borla et al., 2002). The capture swim is initiated only when the larva is within a 3–7° heading of the *Paramecium* and is associated with a peak in the instantaneous tail beat frequency and velocity, and concomitant propulsion of the fish forward to capture the *Paramecium*. It appears to be facilitated by vergence eye movements (Bianco et al., 2011; Patterson et al., 2013). Peak velocity during this capture event is around 30 μm/ms compared to the ubiquitous “slow swims,” which have comparably slower linear velocities of about 15 μm/ms. Predation by larvae thus consists of a series of discrete locomotor maneuvers and is innate in that successful strikes are seen from the first feeding episodes. For juveniles and adults, there are so far only preliminary reports about prey tracking and strike kinematics (Bonaiuto et al., 2007; Westphal and O'Malley, 2010).

### Sensory control of prey capture

In regards to sensory mechanisms of predation, there is accumulating data specifically for larval sensory controls. Larval zebrafish are known to dramatically reduce prey consumption when placed in the dark and do not appear to exhibit the distinct tracking and capture maneuvers used in the light (McElligott and O'Malley, 2005). However, the precise relationship between visual signals and the distinct motor elements of larval prey tracking and capture remain undefined. Gahtan et al. (2005) investigated the role of the optic tectum, (homologous to the mammalian superior colliculus) in the guidance of zebrafish feeding episodes. Bilateral tectal ablations were shown to decrease, but not eliminate, feeding in larval zebrafish (also see Burrill and Easter, 1994, regarding other retinorecipient areas). *Lakritz* mutants, which lack retinal ganglion cells, also show a deficit in feeding rate (Gahtan et al., 2005). More recent data has defined key criteria of the visual stimulus (Bianco et al., 2011; Patterson et al., 2013) and suggested a role for binocular visual information. These data notwithstanding, feeding is *not* abolished in the dark and other sensory modalities may contribute.

The mechanosensory lateral line (Münz, 1989) is a plausible candidate for guiding zebrafish prey capture in the dark, and might also provide short range sensory information to aid predation in circumstances where visual information is readily available. The lateral line is found in fishes and aquatic amphibians and contributes to a variety of behaviors including rheotaxis (Montgomery et al., 1997), eddy chemotaxis (Gardiner and Atema, 2007), station holding (Montgomery et al., 2003), hydrodynamic imaging (Hassan, 1989; Windsor et al., 2008) and predator avoidance (McHenry et al., 2009; Stewart et al., 2013). Of note here is the possible role for lateral line in prey capture (New et al., 2001; Pohlmann et al., 2004), that may derive from its specialized structures. The posterior lateral line consists of an array of neuromasts running along the lateral surfaces of the fish's trunk. This is complemented by an anterior lateral line system comprised of clusters of neuromasts located around the head region.

The further specialization of the lateral line into canal and superficial systems of neuromasts might also facilitate prey detection and tracking. Superficial neuromasts contribute to the detection of water currents moving over the surface of the fish and facilitate the rheotaxic behavioral response (Montgomery et al., 1997; Olszewski et al., 2012). The amplitude of cupula displacement is proportional to the velocity of water flow which enables water velocity to be encoded by the afferent firing rate (Münz, 1989). In contrast, canal neuromasts are located in ossified canals between two pores. When there is a pressure difference at the surface, water flows through the pores, deflecting the neuromast. The canal neuromasts are likely responsible for detection of water vibrations created by a predator (McHenry et al., 2009) or prey item (Montgomery, 1989; Janssen and Corcoran, 1993; Montgomery et al., 2002, 2003; Pohlmann et al., 2004; Bassett et al., 2007). The lateral line (perhaps the anterior neuromasts) was suggested as a possible releaser of the capture swim, but at present there are only preliminary reports on the contribution of lateral line to predation by larval and juvenile zebrafish (Bonaiuto et al., 2007; Westphal and O'Malley, 2010).

### Ontogeny of advanced predatory capabilities

To better understand the ontogeny and neural control of zebrafish predation, more information is needed about the changing kinematics and strategy of predation, as well as the sensory controls that enable this critical behavior. Using high-speed behavioral imaging, we document a rapid increase in the sophistication and efficacy of predatory episodes that is highlighted by (1) a fusion of basic motor maneuvers into seamless “homing strikes” and (2) a surprisingly flexible array of motor patterns (including bend sequences) that allows juvenile fish to effectively strike throughout much of the imaging arena. In addition, we provide evidence that the lateral line system contributes to predatory success across ontogenetic stages.

## Methods

All protocols and experiments were approved by the Northeastern University Institutional Animal Care and Use Committee.

### Zebrafish husbandry

#### Broodstock

Zebrafish adults were maintained at 28–29°C on a light cycle of 14 h light: 10 h dark (lights on at 7 am; lights off at 9 pm). Fish were fed “Omega-One” flake food and *Artemia salina*, each twice per day. Water parameters including temperature, pH, and conductivity were monitored daily. Twenty percent water changes were performed weekly.

#### Larvae

Zebrafish larvae were maintained in an incubator at 28.5°C on the same 14:10 light cycle as adults. Waste was removed and 60–90% of the water was changed daily. Zebrafish were fed *Paramecium multinucleatum* (approximately 200 μm in size) starting at 5 days post fertilization (dpf). *P. multinucleatum* were cultured off site, fresh cultures were delivered weekly. Starting at 10 dpf zebrafish were fed newly-hatched *Artemia salina* nauplii (approximately 450 μm). *A. salina* were cultured daily from cysts (Brine Shrimp Direct). *Paramecium* was continued after the start of *Artemia* until all fish in the clutch displayed a “pink” abdomen, indicating that they were successfully feeding on *Artemia*. All zebrafish used in this study were wild type, of the EKW strain (Ekkwill Water Life Resources, FL).

### Recording of feeding episodes

#### Stages

Because larvae in a given clutch develop at varying rates, total length is a preferred metric to define developmental age (Parichy et al., 2009). Zebrafish were grouped according to total length (TL, in mm), which was used to define the start of successive ontogenetic stages (Figure 1). Fish between 4.0 and 6.0 mm in total length were categorized as early larvae. Fish with a total length between 6.0 mm and 10.0 mm are of the late larval stage. Juvenile fish are between 10.0 mm and 20.0 mm in total length, while fish greater than 20.0 mm in total length are considered adults.

Most feeding videos were acquired at 500 frames/second with a Redlake MotionScope charge coupled device (CCD) camera fitted with a macro zoom lens. Some videos were acquired with a EG&G Reticon high speed camera. To ensure that fish were not satiated prior to observation, food was withheld for approximately 24 h. Larval fish were carefully transferred to a Petri dish with an internal diameter of 34 mm. Juvenile and adult fish were placed in a cylindrical glass bowl with an internal diameter of 102 mm. To minimize movement of the fish within the water column, containers were only filled to about two to three times the height of the fish. During real-time observations, the running 2048-frame buffer of the Redlake camera was manually stopped after feeding was observed and the preceding relevant frames were selected and manually saved for later analysis. A single calibration image of a ruler was taken at the same magnification and saved along with the feeding videos as a calibration frame. This calibration image was used to set the scale (pixels/cm) for all distance measurements.

#### Definitions

The term “feeding episode” is used to describe each entire feeding event and may include both tracking and strike components. It may be one continuous locomotor bout, or may be divided up into “maneuvers” and “pauses.” The “strike” is the last continuous maneuver of a feeding episode, and typically culminates in capture of the prey item.

### Kinematic and behavioral measurements

Videos were viewed frame-by-frame using NIH *Image J*. The following measurements and calculations were made.

#### Statistical analyses

All error bars in the figures represent standard deviation, while the *p*-values in the paper are based upon *t*-tests, including the Welch-Satterthwaite *t*-test, which allows for both unequal sample sizes and unequal variances.

#### Total length of fish (mm)

The length from the tip of the snout to the end of the caudal fin.

#### Duration of feeding episodes (ms)

This was measured from the onset of a feeding episode, i.e., the initial movement towards a prey item, to the moment of capture or contact with the prey item. During visual monitoring of behavior, zebrafish have quiescent periods that are long in relationship to the duration of feeding episodes. To more precisely define the onset of an episode, any active movement that occurs with a gap of 1 s or more earlier than any tracking or strike maneuver leading to prey capture is not considered part of the episode (coasting is considered part of the quiescent periods by this definition). Single maneuver episodes (including adult and most juvenile strikes) occur when there is no active movement within the 1 s window preceding the strike movement bout.

#### Duration of strike (ms)

The strike is the last maneuver of a feeding episode. The start of the strike is marked by the frame preceding the first movement of the final maneuver and terminates when the fish makes contact with the prey item. When the feeding episode is a single, continuous movement bout, that entire movement bout is considered the strike.

#### Distance to prey (mm)

The distance from the tip of the snout to the prey item, measured at any time during the feeding episode.

#### Offset angle (°)

This is the angular deviation between the fish and prey item, measured at any time during the feeding episode. This angle is defined by two lines. The first is a line extended from the midline of the fish at the rostral-caudal location of pectoral fin attachment to the midpoint between the two eyes. The second line is measured from the same midline location of pectoral fin attachment and extends to the center of the prey item.

#### Pause time (ms)

The time during which no axial or pectoral fin movement is observed. The number of pause frames is counted and converted to milliseconds.

#### Cumulative pause time (ms)

The sum of all the individual pause intervals in a single feeding episode.

#### Episode velocity (μm/ms)

This is the straight-line velocity for each complete feeding episode. The distance (in μ m) is measured from the fish's starting position to its final position at the end of the episode (point of contact with the prey item); the distance is divided by the elapsed time (in ms), yielding episode velocity in microns per millisecond.

#### Normalized episode velocity (BL/s)

The normalized velocity is calculated by dividing the episode velocity by the total length of the fish, yielding a velocity measured in body lengths per second.

#### Instantaneous and peak velocity (μm/ms)

Custom MATLAB code was used to track the center of mass of a fish over the entire feeding episode. Videos were first imported into Image J where the threshold function was used to create a black (fish) on a white (background). This was done to minimizes background noise in the frame. The feeding episode was then saved as an image sequence and imported into MATLAB. Once the video was imported, a minimum size threshold was set (70 pixels). This eliminated any background items smaller than the fish. A series of “dilate” commands, followed by a series of “erode” commands were then executed to help ensure continuity of the shape. The series of dilate and erode commands sharpen the borders so that the fish can be tracked. Dilate commands replace the pixel with the darkest neighboring value and erode commands replace the pixel with the lightest neighboring pixel. When performed iteratively it sharpens the edges of the image. The “centroid” command was then used. This command is designed to find the center of area of a 2D shape and so served as a good approximation of the center of mass of the fish. MATLAB then ran through the series of images (.jpg files), recording the *xy* coordinates of the centroid for each frame. These *xy* coordinates and the time elapsed between frames was used to calculate the velocity of the fish. A sliding-window filter averaging *n* = 5 frames was applied. This smoothing function helped to remove any background noise due to slight error inherent in the automated tracking. Manual velocity calculations were also done and these values compared well with those computed in MATLAB. The maximum instantaneous velocity in each episode was recorded as the peak velocity for that episode and these peak values were averaged to determine the mean peak velocity for each group of zebrafish.

#### Normalized peak velocity (BLs/s)

Velocities obtained were normalized to body length by dividing the peak velocity (mm/s) by the total length of the fish (mm). This value is the normalized peak velocity in units of “body lengths per second” (BL/s).

#### Instantaneous tail beat frequency, iTBF

The tail-beat-frequency of forward swimming is normally calculated as the reciprocal of the duration of one cycle of axial bending. The iTBF value is calculated based upon the half-cycle duration. One half cycle starts with a maximum tail bend to one side and ends at the maximum tail bend to the opposite side. The value of 1/(2× half-cycle duration) is equal to the iTBF.

### Sensory manipulations and related analyses

*Paramecia* and *Artemia* were filmed at 250 frames per second for 4 s. Subsequently, videos were analyzed using Image J to determine velocity. Distance traveled between successive frames was divided by the time elapsed to generate instantaneous velocities (μm/ms). The peak instantaneous velocities recorded from 5 individuals were averaged to determine the peak velocity for each species. Average instantaneous velocities were also determined for each individual and averaged over 5 individuals to determine the average velocity for each species.

#### Deprivation of visual information

Sensory information to the visual system was blocked by placing the fish in a light-tight box.

#### Lateral line lesion

By immersing fish in 250 μM neomycin, the lateral line sensory system was disabled (protocol from Owens et al., 2009). Fish were treated for 30 min and then allowed to recover for 1–2 h before experiments were conducted. Behavioral studies were always performed the same day as the neomycin lesion.

#### DASPEI staining

Hair cells were labeled by bath application of the fluorescent mitochondrial dye DASPEI (2-(4-(dimethylamino)styryl)-N-ethylpyridinium iodide) (λ excitation = 461 nm; λ emission = 489 nm) at 0.001% bath concentration for 15 min. Fish were subsequently embedded in 1.2% low melt agarose or 3% methylcellulose and imaged on a BioRad MRC 600 confocal microscope. Successful lesioning of neuromasts after neomycin treatment was confirmed by periodic inspection to observe elimination of the DASPEI labeling. Intact neuromasts appeared as bright, punctate spots on the fish.

#### Feeding success

Ten fish were selected from each of three age groups (8, 24, and 180 dpf) and the percentage of successful strikes recorded. A total of 5 control feeding episodes were collected from each fish. Once control feeding episodes were collected fish were subjected to the lateral line lesioning protocol. After a 1 h recovery period, 5 additional feeding episodes were collected from the same fish. Only those fish with a total of 10 recorded feeding episodes (5 control, 5 neomycin treated) were used in the subsequent analysis.

#### Feeding rates

Zebrafish were individually placed in cylindrical glass bowls (internal diameter of 102 mm, water depth ≈20 mm). *Artemia* were individually counted and added to each dish (30 *Artemia* per dish). Fish were placed into one of four treatment groups: I. Light, lateral line intact; II. Light, lateral line lesioned; III. Dark, lateral line intact; IV. Dark, lateral line lesioned. Fish were allowed to feed for 2 h at which point they were removed and the remaining *Artemia* were counted. The number of *Artemia* consumed in 2 h was used to calculate feeding rate (*Artemia*/hour).

#### Recording of dark feeding episodes

A group of seven 39-dpf zebrafish were placed together in a cylindrical glass bowl (internal diameter = 102 mm) above an infrared light inside a light-tight, dark enclosure. A tube of dark cloth was wrapped around the bowl and extended up to the camera lens providing a second light barrier within the recording box. Videos were collected at 250 frames per second. All events were saved, including both successful and unsuccessful events. At this point, the enclosure was opened and the fabric tube was removed from the dish. An additional recording session yielded 10 control (light) feeding events, including both successful and unsuccessful episodes. Distance at initiation of feeding episodes was measured for both the light and dark conditions.

## Results

Zebrafish do not undergo discrete metamorphic changes, but develop gradually into adult-like form, over a period of several months. To illustrate maturation of form and predatory performance, four successive stages of development were evaluated, namely: early larval, late larval, juvenile and adult. Over the first 30 days, in the progression from early larval into juvenile form, there is a near tripling in length with stream-lining of the body and radical change in fin structure, approaching the adult's caudal fin form. Figure 1 summarizes these changes, showing the typical age at which each stage begins (as defined by starting length). Provided below for these stages are details of strike kinematics, measures of predatory performance, and sensory system contributions.

**Figure 2:**
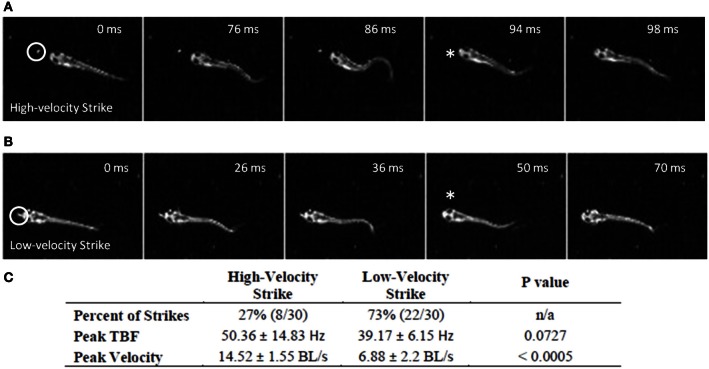
**Early larval prey strikes vary in locomotor speed**. In **(A)** there is pronounced bending of the caudal trunk which results in abrupt acceleration through the prey item. The *Paramecium* is circled in the first frame, while the asterisk marks the moment of capture. **(B)** Also shows a caudal bend and acceleration, but not to the same degree as **(A)**. In early larvae, these strikes (capture swims) typically follow a series of tracking maneuvers that align the larva with the *Paramecium*. Larvae shown in **(A)** and **(B)** are 4.1 mm in length. **(C)** Summarizes the peak tail-beat frequency and velocity values of the low and high velocity strikes.

While early larval predatory episodes were comprised of discrete tracking maneuvers and a brief capture swim as previously reported, variations were noticed. A minority of strikes exhibited especially pronounced bending of the caudal trunk (Figure 2A), while subtler bending was more typical (Figure 2B). The greater bending was associated with faster velocity and a slightly higher tail beat frequency (Figure 2C), leading to more abrupt acceleration through the prey item. These episodes all fell, nonetheless, within the general locomotor pattern previously described for early larval capture swims. Young larval zebrafish sometimes consume prey by stationary suction feeding (Budick and O'Malley, 2000), which continues at later developmental stages, but most predatory episodes observed in these experiments involved substantial locomotion. Because locomotor aspects of predation were of interest, the data below concern only feeding episodes with a significant locomotor component.

**Figure 3:**
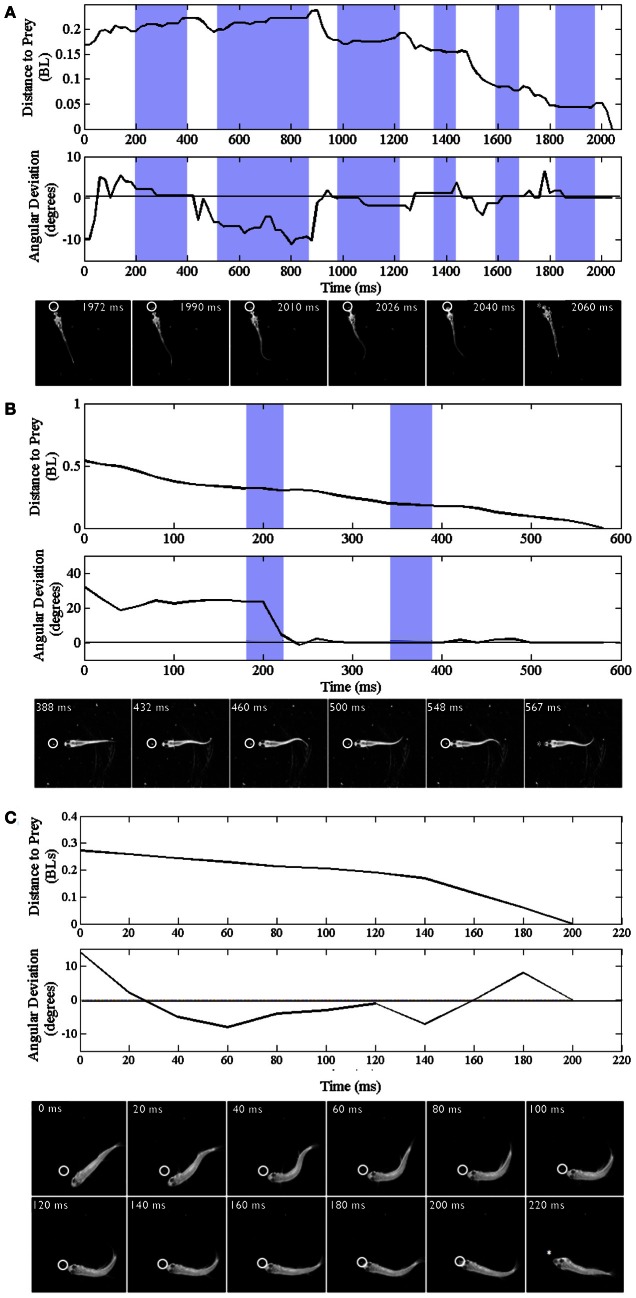
**Trajectories of early larval, late larval and juvenile feeding episodes**. Three representative episodes are illustrated via plots of distance to the prey item (upper plots) and changes in offset angle (in degrees), between the fish and prey item (lower plots). The montages of frames from the high-speed videos illustrate locomotor components of just the final strike; all times are in relation to the initial movement towards the prey item (*t* = 0). Shaded portions of the plots represent pauses in locomotor movements, whereas non-shaded areas represent active swimming or turning. The final capture swim or strike occurs in the last non-shaded segment of each plot. **(A–C)** Represent early larval, late larval and juvenile episodes respectively. **(A)** Representative episode of an early larval zebrafish (3.8 mm in length) feeding on a *Paramecium*; total episode duration was 2024 ms. In this episode the tracking phase includes one J-turn (the first non-shaded area) and five forward swimming maneuvers. This is followed by the final maneuver, a strike (capture swim) as illustrated in the montage. **(B)** Feeding episode of a representative late larval zebrafish (9.2 mm) was 576 ms in duration. It included two tracking maneuvers, two pauses (shaded areas), with the final strike consisting of rhythmic trunk bending as seen in the montage. **(C)** Example juvenile feeding episode has no pauses and shows continuous closing and aligning to the prey before the final strike, which occurred 200 ms after the initial turn towards the *Artemia*.

Trajectories are presented for zebrafish stages along with montages of kinematic features of the final strike (Figure 3); all times shown are in relation to initiation of a feeding episode (at 0 ms). Early larvae (Figure 3A) initiate feeding episodes at a relatively short distance from the target prey item (a *Paramecium*), typically less than one half the fish's own body length. The tracking phase consists of distinct J-turns and forward slow swims, separated by brief pauses, and is followed by a distinct capture swim bout. In a subsequent, transitional phase, the late larval stage, predatory episodes are varied in nature, but often comprised of multiple movements. The example in Figure 3B has two pauses and just two tracking maneuvers that align the larva with the *Artemia*; it is also shorter in duration. The segregation of function into distinct kinds of tracking maneuvers becomes blurred, in this transition, as does the distinction between tracking and strike maneuvers. While some elements of early larval tracking are retained by the late larvae, more complex maneuvers are frequently observed in which forward propulsive and turning movements are conjoined, resulting in distinct trunk kinematics.

### Fusion of locomotor elements

In the example juvenile feeding episode shown (Figure 3C), the juvenile moves continuously from episode initiation through to striking the *Artemia*. Absent pauses, the total elapsed times of juvenile feeding episodes can be much smaller (note the different time scales in Figures 3A–C). Also, the example of Figure 3C has no substantive “forward swimming” component or rhythmic trunk bending. Instead, after a caudal kink in the trunk at 20 ms, there is a pronounced bend to the right that helps propel the juvenile through the *Artemia*'*s* location. This is but one example and juvenile strike patterns are quite varied as described below.

Replay of high-speed video of prey capture episodes allowed them to be classified as single maneuver, or multi-maneuver (using discrete movement bouts separated by pauses). For instance, a 5-day-old larval episode might consist of: J-turn – pause – slow swim – pause – capture swim. As zebrafish mature, the discrete tracking and strike maneuvers used by early larvae disappear. Most juvenile and all adult zebrafish instead display a single, continuous (and often complex) feeding maneuver where orientation and approach to a prey item are merged together. Because these fluid maneuvers often cover significant distance and/or show substantial direction change, they are referred to here as “homing strikes.” In quantitative terms, all early larval feeding episodes (*n* = 43) contain multiple movement bouts, while 73% of late larval feeding episodes (*n* = 37) used two or more maneuvers and only 24% of juvenile episodes (*n* = 34) were multi-maneuver. Thus, the large majority of juvenile and all adult locomotor-driven feeding episodes (*n* = 38) were single maneuver episodes, i.e., homing strikes.

### Predator vs. prey

**Figure 4:**
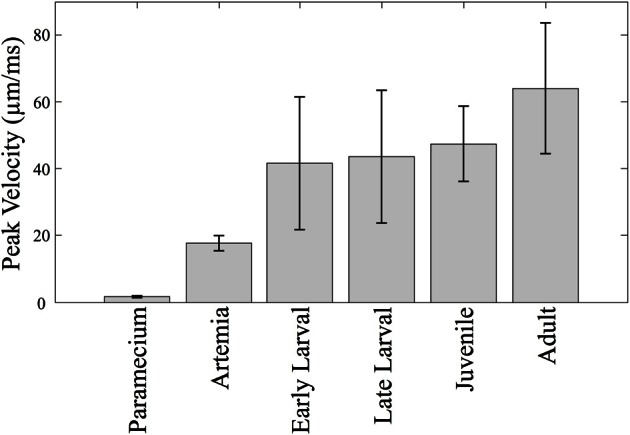
**Predator vs. prey velocities**. The mean peak velocity of different prey items are shown along with velocities of zebrafish at different stages. The mean peak velocities of the zebrafish increase over time, but not dramatically so. Zebrafish of all ages are much faster than *Paramecia* and *Artemia*.

Relative swim velocity is important in that faster velocities may enable capture of faster prey. Figure 4 summarizes mean peak velocity (in μm/ms) of the main prey items studied here, *Paramecia* and *Artemia*, along with the zebrafish mean peak velocities which, at all stages, are higher than the prey items consumed during those stages. Early larvae show peak velocities ranging from 22 to 79 μm/ms, much faster than *Paramecia* which swim at an average velocity of 0.61 ± 0.1 μm/ms s.d., and a peak velocity of 1.72 ± 0.3 μm/ms, s.d. *Artemia* swim at an average velocity of 5.19 ± 0.7 μm/ms s.d., and a peak velocity of 17.6 ± 2.3 μm/ms, s.d. *Artemia* are preyed upon by late larval and juvenile zebrafish, which have peak velocities ranging from 21 to 89 μm/ms and 32 to 94 μm/ms, respectively. Zebrafish can thus attain peak velocities that are much faster than these laboratory prey items.

### Measures related to predatory capability

**Figure 5:**
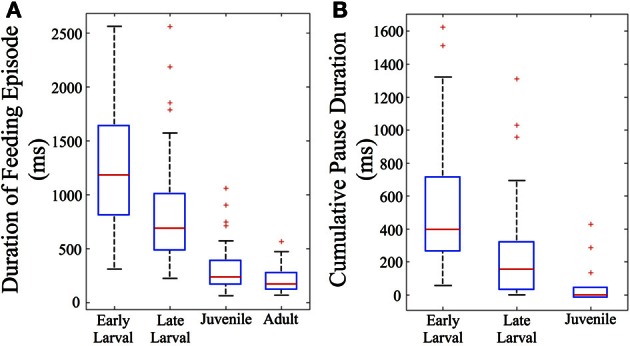
**Feeding episodes become shorter and pause time decreases across ontogeny**. Feeding episodes were binned according to ontogenetic stages (based on total length) and the durations and total pause time determined. In the box plots used to represent these data, the central red line indicates the median value of each data bin, while the 25th and 75th quartiles are enclosed inside the box profiles. Note that mean values in the Results are higher than the median values due to the effect of one-sided, high numerical value outlier data points, shown as red crosses. **(A)** The duration of feeding episodes (including pauses and maneuvers) falls across ontogeny (*n* = 156). **(B)** The summed duration of the individual pauses between discrete motor elements also falls across ontogeny (*n* = 82). By definition, only multi-maneuver feeding episodes include pauses; there are no pause data for adult fish which were always “single-maneuver.”

The total duration of individual feeding episodes (which includes tracking and capture components) decreases over ontogeny (Figure 5A). Early larval feeding episodes are the longest, and duration falls dramatically by the juvenile and adult stages. The early and late larval episodes were significantly longer than juvenile and adult feeding episodes (*p* < 0.005), demonstrating that older fish are able to capture prey items more quickly (at least within the constraints of our feeding arena). Also calculated was cumulative pause time for all episodes that included multiple maneuvers (*n* = 82). This fell sharply with development (Figure 5B). The early larval cumulative pause durations (mean = 534 ± 402 ms, s.d.), were much longer than those of both late larval (326 ± 329 ms, s.d.) and juvenile (115 ± 146 ms, s.d.) fish. Because adults were never observed to pause during individual feeding episodes, they are not represented in Figure 5B.

**Figure 6:**
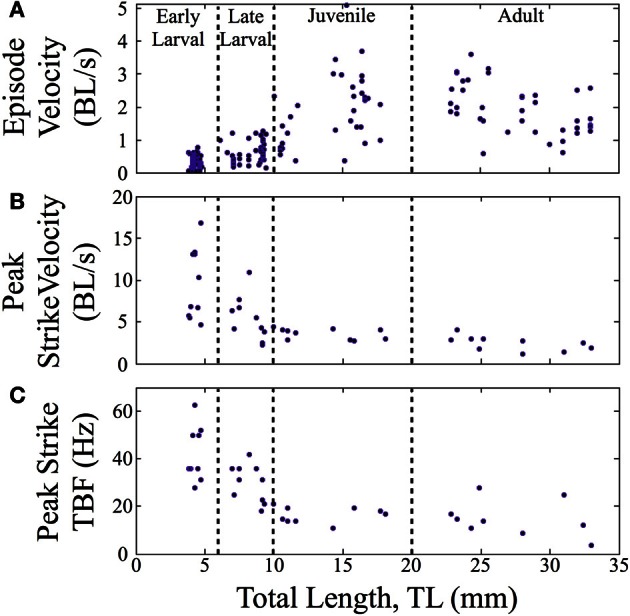
**Normalized feeding velocities and tail beat frequency change across ontogeny**. Scatter plots are shown with the successive stages separated by vertical lines. **(A)** The normalized episode velocity (in body lengths/s) of feeding episodes increases over time, which is due at least in part to decreasing pause time. **(B)** Early larvae show the highest peak velocities, which occur at the end of the capture swim bout. Peak velocity then declines as zebrafish transition through the late larval stage and become juveniles; any further decline is slight. For early larvae, the strike is a tiny fraction of the feeding episodes, hence the large peak values in **(B)**, but low episode velocities in **(A)**. **(C)** Early larvae also show a high peak tail-beat frequency, which then declines across subsequent stages in a pattern mirroring the decline in peak strike velocity.

In terms of swimming speed, both peak strike velocity and episode velocity (average speed across an entire episode) were measured. Normalized episode velocity, which includes any pause time, increased over ontogeny (*n* = 157, Figure 6A), primarily in the late larval to juvenile transition. The data points for early larvae (4 to 6 mm TL) are fairly clustered, in contrast to the broader distributions for older fish. A subset of feeding episodes (*n* = 40), of sufficient quality for automated analysis, was further analyzed to determine the normalized peak velocity and peak tail beat frequency for feeding strikes. An exponential decrease in normalized peak velocity (BL/s) is seen as fish mature (Figure 6B). Concurrently, there is a sharp decline in peak tail beat frequency (measured in Hz; Figure 6C), indicating a change in the operation of neural oscillators in spinal cord.

**Figure 7:**
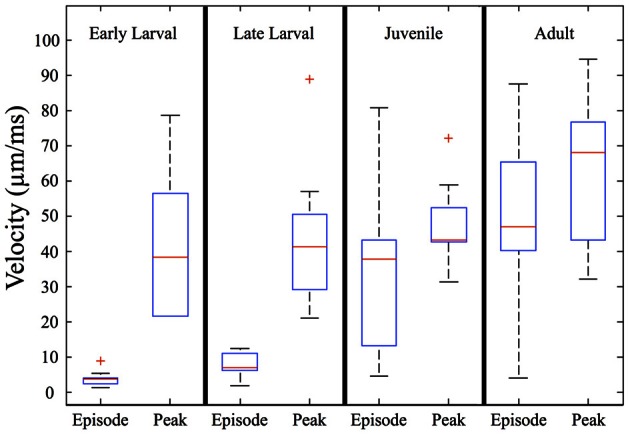
**Episode velocity increases across the larval to juvenile transition**. The episode velocity (average velocity across each feeding episode) increases markedly from the larval to juvenile stages. In contrast, the peak absolute velocity does not change greatly, but trends larger for adults. The differences between peak and episode velocity are significant for both early larvae and late larvae (*p* < 0.0005), which is explained by an abrupt peak occurring during capture swims. Because the juveniles and adults mostly lack pauses, their episode and peak velocities are more similar, yet still show transient peaks during their predatory maneuvers. In addition, the larval episode values are tightly clustered, but become highly variable in the juvenile and adult stages, reflecting more diverse predatory behaviors. Red crosses represent outlier data points.

When measured in absolute terms (μm/ms), early larvae were (somewhat unexpectedly) seen to exhibit absolute peak swimming velocities roughly comparable to those of juvenile and even adult zebrafish (Figure 7), although adults showed the highest recorded velocities. In contrast, the overall episode velocity increased dramatically, especially across the larval to juvenile transition. These results are explained by the pronounced but brief burst of acceleration seen in early larval capture swims which yield high peak velocities, whereas episode velocities are low due to the frequent pauses. The differences between episode vs. peak velocity are statistically significant for both early and late larvae (*p* < 0.0005).

**Figure 8:**
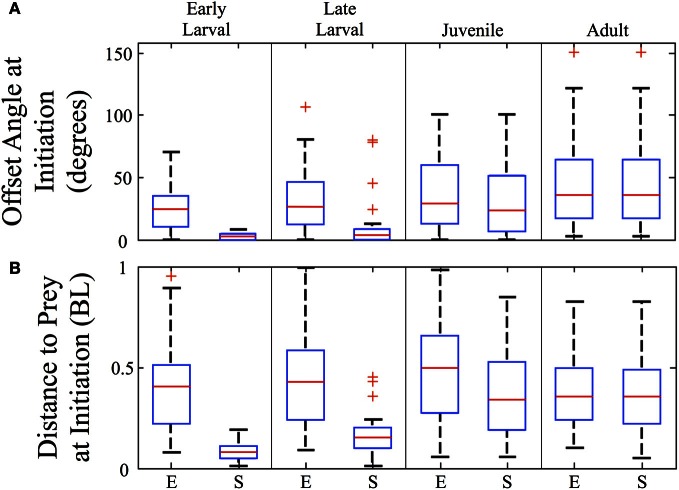
**Strike offset angle and distance increase sharply across development**. Box plots are shown for each ontogenetic stage providing the offset angle **(A)** and the normalized distance **(B)**, in body lengths (BL) at the initiation of both the feeding episode (E) and the final strikes (S). The strike offset angles and strike distances show substantial increases, particularly at the larval to juvenile transition. In contrast, the overall (episode) angle from which predation is launched increases only modestly, while the normalized overall (episode) distance does not show a consistent trend. Red crosses represent outlier data points.

The angles and distances from which feeding episodes are launched are another measure of predatory capability. Figure 8A shows the offset angles from which both episodes (E) and final strikes (S) are launched (*n* = 157). Offset is the angle between the zebrafish's orientation and the direction to the prey item, so that a 90° offset angle would require a net right or left turn to approach the prey. The strike offset angle is miniscule for early larvae, but increases markedly across development. In contrast, episode offset angles begin large and increase further. For both early and late larvae, the offset angles are significantly smaller for strikes than episodes (*p* < 0.005). By the adult stage, all feeding episodes are comprised of single movement bouts and so the episode and strike offset angles are essentially the same and represent an ability to strike throughout much of the feeding arena. As the box plots indicate, zebrafish of all ages tend to initiate predatory episodes within ±60° of their heading. In regards to strike distance, Figure 8B shows that strikes (S) are launched from very close distances in early larvae, but this rises many fold in juveniles and adults, even as normalized to body length. Feeding episodes (E), however, are launched at similar (normalized) distances across age groups.

### Diversity of juvenile homing strikes

**Figure 9:**
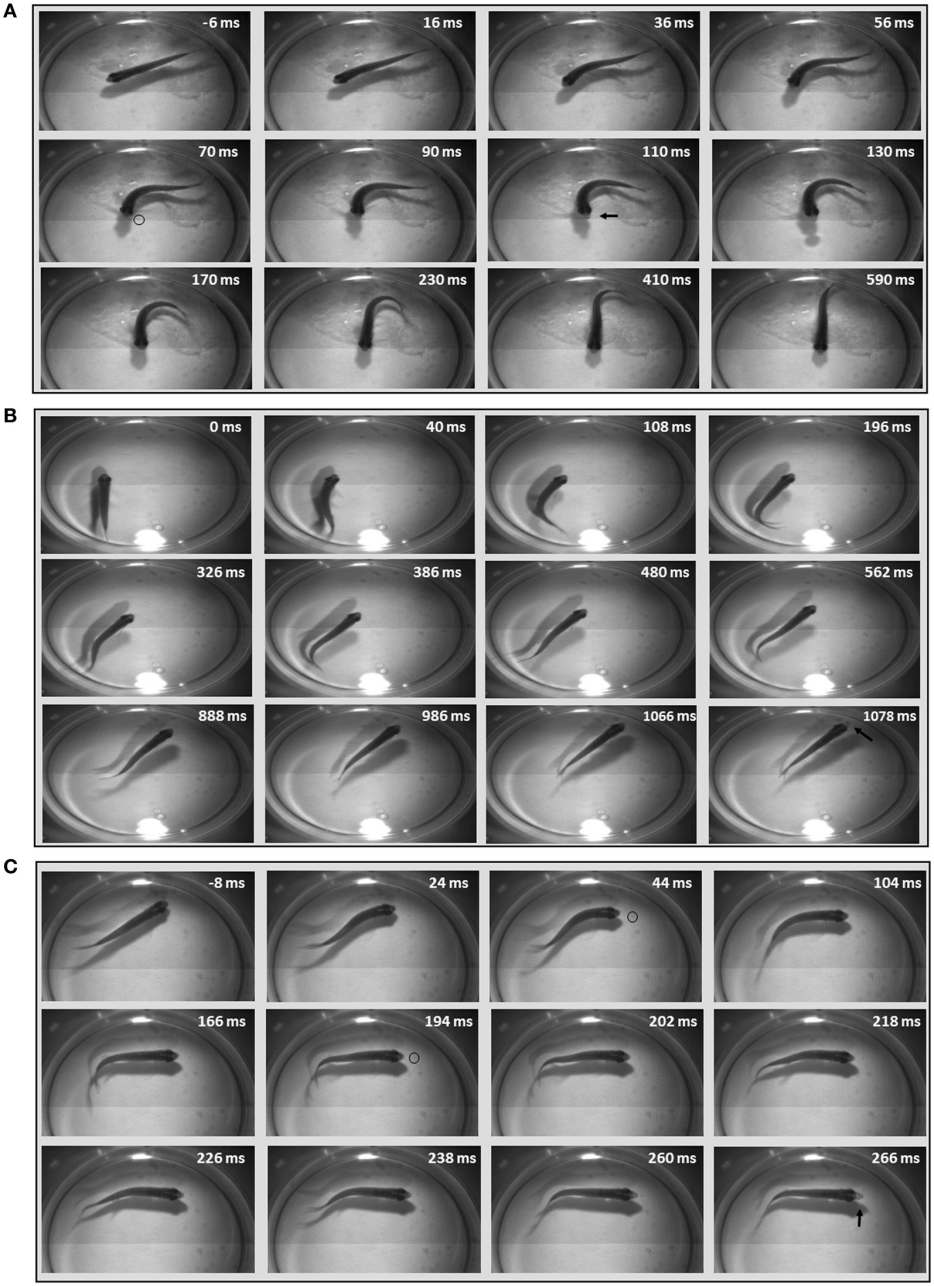
**Diversity of juvenile homing strikes**. Three example homing strikes were chosen to represent the diversity of kinematic patterns and motor commands used in juvenile strikes. Select frames are shown with elapsed time after initiation of episode given in milliseconds; negative numbers denote resting frames before the strike. The first strike example **(A)** shows a single large bend, with prey capture occurring as the head swings to the *Artemia*. *Artemia* are easier to see in movies than still images, but the small dot (inside black circle) is an *Artemia* nearing the juvenile's mouth, at 70 ms. It is evident in the next frame at 90 ms, but is obscured (or gone) in the next frame, where the lightly colored jaw is protruding (indicated by arrow) at 110 ms. **(B)** Shows rhythmic, slow forward swimming after an initial turn. Bending movements stop near the end of the episode, before the juvenile coasts through the *Artemia*'*s* location (which is very faint). Jaw protrusion can be seen in the last frame (arrow). **(C)** Shows a more elaborate maneuver, with details on the bend sequence described in the text. The location of the *Artemia* is slightly more visible in these still images and is marked at several time points by black circles. Jaw protrusion is most pronounced in the final frame (arrow).

Figure 9 illustrates the diversity of strike patterns used by juvenile zebrafish. These early data were collected in a smaller, relatively confined feeding arena that may have imposed greater demands in terms of striking at prey items. The three exemplar strikes all consist of a single bout of continuous trunk movement, with precise orienting turns and propulsion executed in close coordination with jaw protrusions (arrows), hence the term “homing strikes.” The first example (Figure 9A) consists of a single large but slow bend that precisely orients the juvenile towards the *Artemia* (inside circle). There are no subsequent bends, so this strike lacks the rhythmic component of escape and slow swimming behaviors. In contrast, the homing strike in Figure 9B consists of an initial turn followed by five cycles of slow swimming, after which the larva coasts directly through the *Artemia*'*s* location.

The last example (Figure 9C) documents more flexible regulation of axial musculature. This homing strike consists of three bends in total beginning with a large, sustained and rostral bend (turn) that orients the rostral trunk towards the *Artemia*. Two smaller, caudal bends are superimposed upon the rostral bend and seem to propel the well-oriented rostral trunk directly towards the *Artemia*. This particular strategy seems related to the close proximity of the Artemia which could be seen swimming near the right eye of the juvenile, eventually reaching a suitable position for a strike to be launched (black circles indicate *Artemia*'*s* location at different times). Jaw protrusions and *Artemia* are more readily observed when viewing movies, versus still photos, but the jaw protrusion (arrow) in the last frame of Figure 9C is evident and emerges at the proper time for efficient prey capture. The kinematic features of these exemplar homing strikes differ sufficiently from one another as to be considered distinct predatory strategies.

### Contributions and maturation of sensory systems

**Figure 10:**
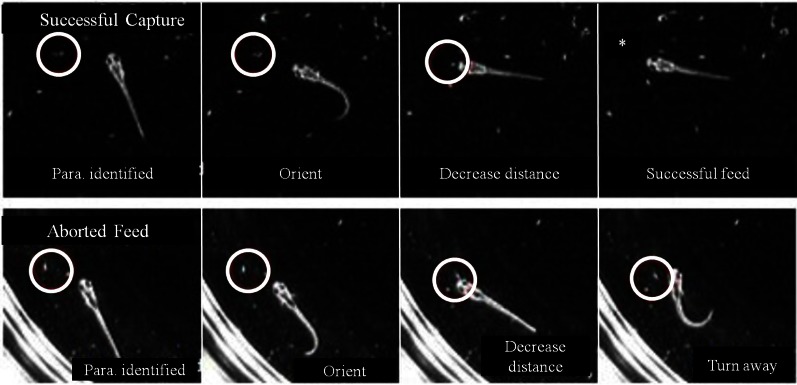
**Aborted vs. successful feeding episodes of larval zebrafish**. Tracking maneuvers appear similar between successful feeding episodes (**upper panel**) and aborted feeding episodes (**lower panel**). Both panels show the onset of tracking, orientation to the prey and a closing maneuver to the point where the larvae are very close and well oriented towards the *Paramecium*. While the larva successfully engulfs the *Paramecium* using a capture swim in the upper sequence, the aborted episode in the lower sequence shows a distinct turn that produces a large offset angle away from the prey item, ending the episode (the edge of the Petri dish is seen in the **lower panel**).

In some first feeding episodes, young larvae oriented and moved towards a nearby prey item, but did not consume it. Failed episodes resulted from both misses and, more frequently, aborted episodes, where items were tracked and approached, but no strike was launched. In comparing successful and aborted episodes (Figure 10), both showed larvae orienting via J-turns towards a *Paramecium* and approaching it, but in the aborted case (lower row), the larva abruptly turned away terminating the feeding episode. Table 1 provides a comparison of 19 successful and 19 aborted feeding episodes.

**Table 1:** **Completed vs. Aborted feeding episodes**.

	**Completed feeding episode**	**Aborted feeding episode**	***p*-value**
Episode duration	1138 ± 567 ms	1684 ± 780 ms	0.0149
Cumulative pause time, %	39 ± 16	55 ± 22	0.0142
Average prey velocity	0.94 ± 0.48 mm/s	0.87 ± 0.6 mm/s	0.2672
Distance at start of episode	1.73 ± 0.93 mm	1.37 ± 0.78 mm	0.113
Distance, final pause onset	0.57 ± 0.53 mm	0.36 ± 0.22 mm	0.17
Duration of final pause	58.42 ± 51.11 ms	494.38 ± 313.87 ms	<0.0005
Prey movement, final pause	0.05 ± 0.05 mm	0.34 ± 0.36 mm	<0.0005
Prey velocity, final pause	0.74 ± 1.2 mm/s	0.76 ± 0.32 mm/s	0.9
Distance at strike/abort	0.34 ± 0.18 mm	0.77 ± 0.59 mm	0.0123

Distances and times associated with successful and aborted feeding episodes are provided. Pause time is given as a percentage of the total duration of each feeding episode.

The decision of early larval fish to abort a feeding episode did not correlate with either prey velocity or distance to prey at strike initiation/abort (in the aborted trials, the prey were slightly closer). Aborted efforts had only modestly longer episode durations, but the final pause duration, after which the larva either strikes at the *Paramecia* or turns away, showed a large difference: the final pause of aborted episodes was 8-fold longer than those seen in successful episodes. The cause of aborted episodes is unknown, but may have to do with the sensory detection of prey items, as considered next.

**Figure 11:**
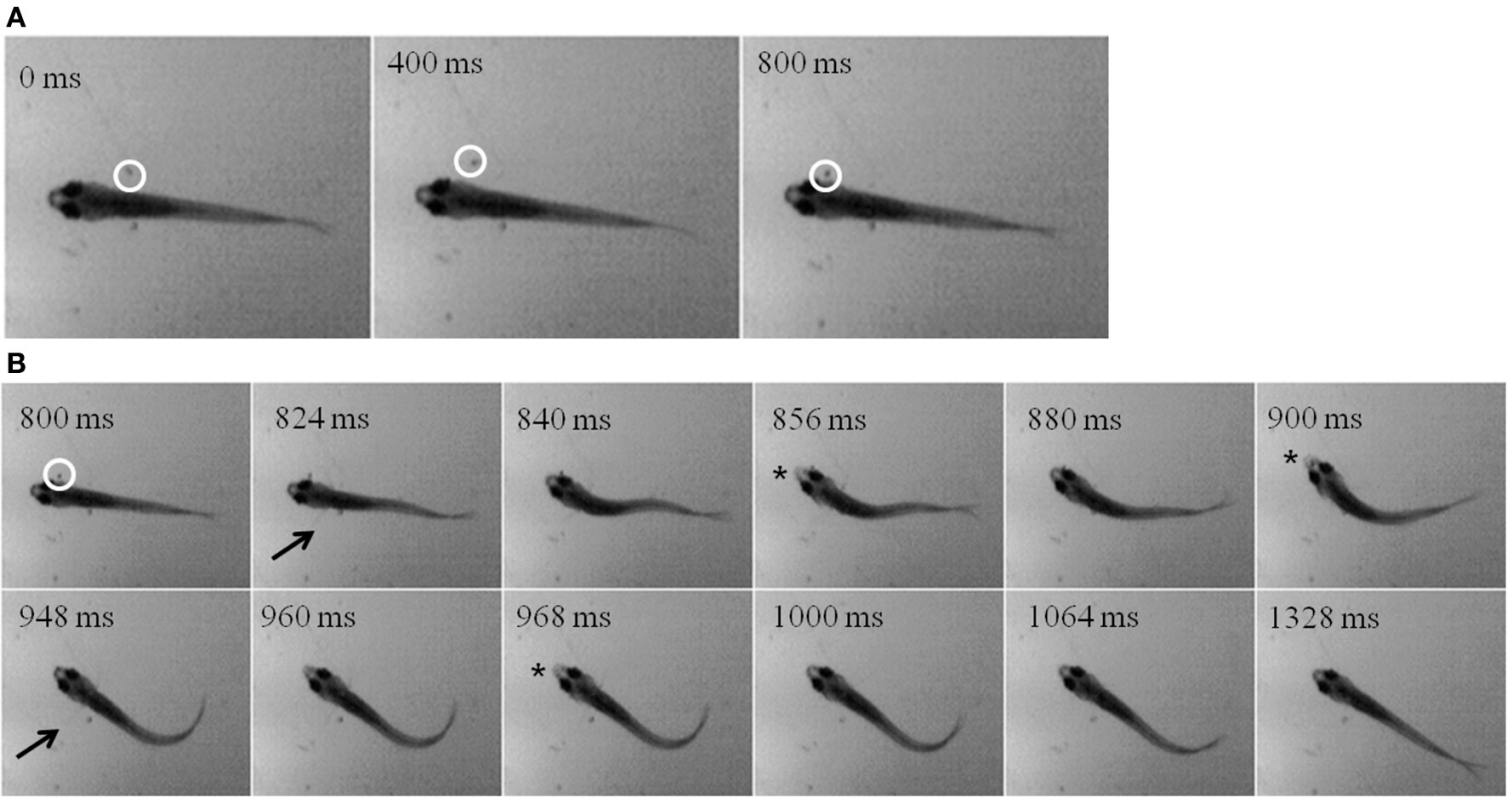
**“Dark Feeding” episode of a 39 dpf, juvenile zebrafish**. Video was collected at 250 frames per second under IR-illumination and representative frames are shown. **(A)** Three frames, each separated by 400 ms, show the motion of the prey item which occurs before initiation of the feeding episode. Note that the *Artemia* is moving in a caudal to rostral direction as highlighted by the white circle. **(B)** Dark feeding by this juvenile is documented via select frames showing locomotor (fin) movements (arrows) and strike attempts, i.e., jaw protrusions (asterisks), with success occurring on the third attempt. The fins are very thin and light and so difficult to see in still photos, but are more apparent when viewing movie files. Note that the jaw protrusion occurring at 968 ms can be seen as a light, anterior extension of the snout, which is not occurring in the frame just above it (at 840 ms).

While zebrafish are primarily visual predators, they do feed in the dark, albeit at lower rates. Using infrared (IR) illumination, zebrafish and prey movements were recorded in the dark. A representative episode is shown in the montages in Figure 11, where an *Artemia* (inside white circle) can be seen swimming alongside a juvenile for about 800 ms (Figure 11A). Then, the zebrafish abruptly turns and makes several attempts to capture the *Artemia*, beginning about the second frame in Figure 11B (pectoral fin movements are indicated by arrows). Two unsuccessful strikes (indicated by jaw protrusions, asterisks) occur at 856 and 900 ms, while a third strike at 968 ms was successful.

The mechanosensory lateral line is a candidate system for mediating prey capture in the dark. To investigate this possibility, feeding rates of late larval (*n* = 96) and juvenile (*n* = 68) zebrafish (that had not been fed for 24 h) were measured under four different sensory conditions: (a) Light with lateral line intact; (b) Light with lateral line lesioned (by neomycin exposure); (c) Dark with lateral line intact; (d) Dark with lateral line lesioned. After housing fish with 30 *Artemia* for 2 h, the remaining *Artemia* were counted (Table 2). Both late larvae and juveniles consumed far more *Artemia* in the light, as compared to dark. In the “Light + Neomycin” group, feeding rates were not significantly depressed, but the combination of “Dark + Neomycin” leads to dramatically lower feeding rates, with larvae consuming about 37-fold fewer *Artemia* than during normal feeding in the light.

**Table 2:** **Feeding rates of late larval and juvenile zebrafish in the presence and absence of visual and lateral line sensory information**.

**Treatment**	**Feeding rate (artemia/2 h)**	
	**Late larvae (***n*** = **96**)**	**Juveniles (***n*** = **68**)**
Light	16.92 ± 9.57	29.96 ± 0.21
Light + Neomycin	12.33 ± 10.26	27.86 ± 3.67
Dark	2.70 ± 5.56	8.88 ± 9.73
Dark + Neomycin	0.45 ± 1.0	3.2 ± 3.11

**Figure 12:**
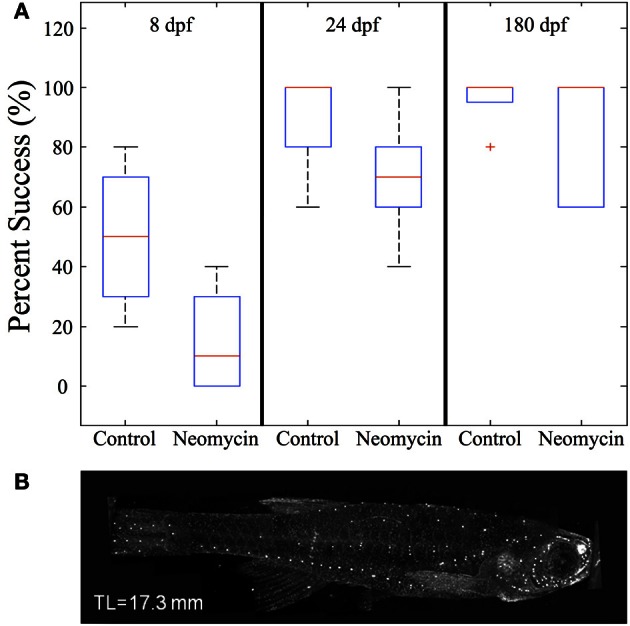
**The effect of neomycin exposure on feeding success in zebrafish. (A)** While early larvae are able to feed on *Artemia*, at 8 dpf their strikes are successful only about 50% of the time and this success rate is less after neomycin treatment. Both 24 dpf fish and adult zebrafish are quite efficient in capturing *Artemia* in the light. Success rate in 24 dpf fish is significantly degraded by neomycin, although the success rate is still greater than with 8 dpf fish. (8 dpf *n* = 4; 24 dpf *n* = 8, 180 dpf *n* = 5). **(B)** Shows DASPEI labeling of anterior neuromasts on an older juvenile zebrafish. Neomycin treatment eliminates neuromasts (and this labeling) for a day or more (Owens et al., 2009). The red cross represents an outlier data point.

To further investigate the impact of lateral line lesioning, high-speed recordings were made of normal and neomycin-treated fish. The average value of feeding success (given as the percentage of successful strikes) was lower for all age groups after neomycin exposure (Figure 12A). The drop in feeding success in the late larval, 24 dpf group was statistically significant (*p* < 0.05). The median success rate of early (8 dpf) larvae also dropped, from 50% to about 10%, and while this result was not statistically significant, some of these larvae captured zero *Artemia*. Early larvae have a more difficult time with *Artemia* (vs. *Paramecia*) because of their size, but the drop-off in success rate with neomycin suggests a role for lateral line in guiding the capture swim, which is potentially mediated by anterior neuromasts, shown stained with DASPEI in Figure 12B. The adult fish were most successful with many adults showing 100% success in both the control and neomycin groups.

**Figure 13:**
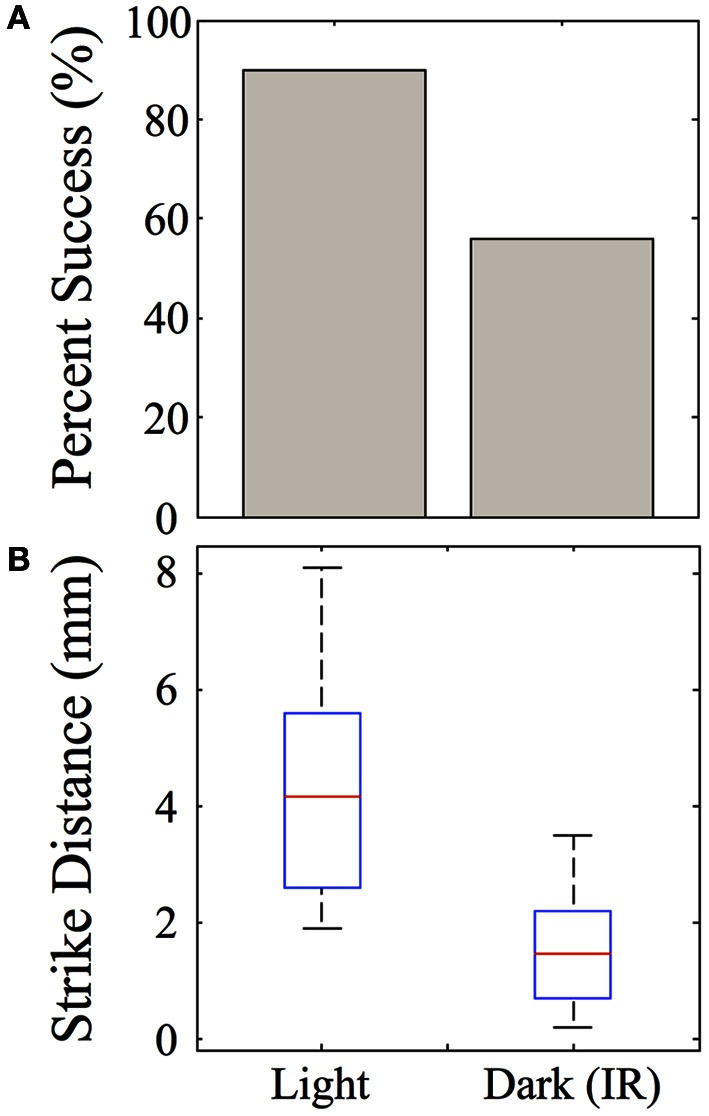
**Effect of darkness on juvenile feeding success and strike distance**. Juveniles were observed striking at Artemia under IR illumination and this behavior was compared with feeding strikes made in the light. **(A)** The success rate of strikes on *Artemia* are roughly 90% in the light, but substantially lower in the dark for these 39-day-old juveniles. **(B)** Strikes made in the dark are initiated from shorter distances than those in the light *(t-test, p = 0.02)*, which fits with the suggested involvement of lateral line. *n* = 9 fish.

Strike distances and success rates were compared in light vs. dark conditions, as shown for feeding episodes of nine 39-dpf juvenile zebrafish recorded under IR vs. normal illumination (Figure 13A). Of nine feeding events recorded in the dark, five were successful (55%), compared with 9/10 (90%) successful feeding episodes from the same fish in the light. During dark feeding episodes, juveniles initiated feeding at 1.55 ± 1.11 mm from the prey, but launched strikes from 4.29 ± 1.96 mm in the light (*p*-value = 0.02) (Figure 13B). This fits with the longer range of vision vs. lateral line, which functions better at short distances. While sustained tracking movements were not apparent in the dark, in agreement with data of Patterson et al. (2013) on larvae, our modest set of recorded dark feeds precludes strong conclusions on juveniles.

## Discussion

The transition of zebrafish predation from discrete, simple maneuvers to elegant homing strikes is suggestive of an evolutionary process whereby progressively more sophisticated motor programs emerged from basic motor patterns. The observed ontogenetic progression can be considered from three distinct contexts: improving motor performance, sensory contributions and the underlying neural controls.

### Locomotor performance

The predatory capabilities of early larval zebrafish are limited but presumably sufficient for natural prey items, although ecological details remain sparse (Engeszer et al., 2007a,b; Spence et al., 2007, 2008). Over the next 4 weeks, motor performance improves rapidly and is highlighted by the fusion of discrete early larval maneuvers into seamless homing strikes. This is not a simple “merger” of tracking and capture movements because J-turns and the kinematic features of capture swims disappear. Instead, what emerges is an ability to fuse orientation and propulsion, which allows juveniles to strike prey items over a wide range of angles and distances within the arena (Figure 8). This fusion of tracking and capture maneuvers also enables a sharp drop in feeding episode duration (Figure 5), while the episode velocity increases in both normalized and absolute terms (Figures 6, 7). In contrast, early larvae are able to launch their *final strike* only when they are very close and precisely aligned with the prey (Figure 8), although *feeding episodes* are initiated from much larger directions and distances.

This is not to discount early larval efforts, which are quite successful from first feedings, but rather reflects changing capabilities as zebrafish grow in size and begin to strike larger and faster prey items. The observed peak feeding velocities of larval and juvenile zebrafish do not change greatly across ontogeny, in absolute terms (Figure 7), although they exceed the velocity of our laboratory prey items, *Paramecia* and *Artemia* (Figure 4). What seems more important are the shorter overall durations of episodes: faster relative velocities may often be useful in predation, but a more rapid rate of capture should be advantageous in terms of total acquired nutrition and in terms of competition with conspecifics, if resources are limited.

### Sensory contributions to prey capture

Many fishes visually hunt prey items (Drost, 1987; Batty et al., 1990; Job and Bellwood, 1996; New et al., 2001; Rice and Westneat, 2005; Fleisch and Neuhauss, 2006) and vision is central to larval zebrafish feeding, based on the role of optic tectum (Gahtan et al., 2005), the lack of tracking movements in the dark (McElligott and O'Malley, 2005; Patterson et al., 2013) and the elicitation of J-turns by artificial visual stimuli (Bianco et al., 2011). Vision remains the predominant sensory modality in older larvae and juveniles, given the large drop in feeding rates seen in the dark (Table 2) and the longer strike distance and better accuracy in light vs. dark conditions (Figure 13). Feeding continues, however, at a low rate in darkness and juveniles can execute targeted predatory maneuvers (Figure 11), suggesting use of another modality for prey detection.

Lateral line contributes to predation in a variety of fishes (Montgomery, 1989; New et al., 2001; Pohlmann et al., 2004). Table 2 shows that ablation of lateral line sensors with neomycin decreases feeding rate in both late larvae and juveniles, but not to nearly the extent as darkness. Neomycin also decreases strike accuracy in the light, in both early and late larvae (Figure 12), but older juveniles fare better, perhaps because visual targeting and/or inertia of the strikes makes the lateral line less necessary. The combination of darkness and neomycin has the most profound effect, reducing feeding to very low levels in late larvae and juveniles (Table 2). Early larvae strike (initiate capture swims) from very close range (Figure 8B), and anterior neuromasts (visualized in Figure 12) may aid this behavior (New et al., 2001; Pohlmann et al., 2004). Our neomycin data, however, are not definitive regarding capture swim modality because these swims remain well formed in the *Light + Neomycin* condition. Contributions to the control of capture swims by olfactory and gustatory senses (Caprio, 1978; Kanwal and Finger, 1997; Friedrich et al., 2004; Gardiner and Atema, 2007) or tactile interactions (Patterson et al., 2013) remain a possibility.

Optic tectum is the largest visual structure in the teleost brain (Burrill and Easter, 1994; Wullimann et al., 1996), and accumulating evidence suggests a role in zebrafish prey capture (Gahtan et al., 2005; Del Bene et al., 2010; Bianco et al., 2011). The larval optic tectum already has a diversity of cell types (Niell and Smith, 2005; Sumbre et al., 2008; Gabriel et al., 2012) which is potentially greater by the juvenile stage and might contribute to the visual analyses that guide homing strikes. Tectum appears to play a central role in sensorimotor transformations that convert spatial information into motor commands (Ewert, 1987; Scott and Baier, 2009; Ahrens et al., 2012; Grama and Engert, 2012), presumably in conjunction with brainstem circuits and associated descending pathways that include the reticulospinal array and nMLF (Lee and Eaton, 1991; Foreman and Eaton, 1993; O'Malley et al., 1996; Zelenin et al., 2001; Bosch and Roberts, 2001; Gahtan et al., 2002; Gahtan and O'Malley, 2003; Sankrithi and O'Malley, 2010). Details of these transformations are just beginning to be understood (Del Bene et al., 2010; Bianco et al., 2011; Koyama et al., 2011; Fajardo et al., 2013).

### Underlying neural controls

Larval J-turns require neural controls distinct from other larval turns being (1) of much slower angular velocity than escape turns (Eaton et al., 1991; Liu and Fetcho, 1999) and (2) kinematically distinct from spontaneous routine turns (Budick and O'Malley, 2000; Danos and Lauder, 2007) which are also used in optomotor behaviors (Roeser and Baier, 2003; Day et al., 2006; Burgess and Granato, 2007; Orger et al., 2008). J-turns are incremental “turn left” or “turn right” responses (Bianco et al., 2011) that achieve precise orientation in a stepwise fashion. The significant pauses between discrete tracking maneuvers (Figures 3, 5) might allow for visual updating of direction and distance, after which the correct next maneuver can be selected. Recent optogenetic results suggest there are specialized tectal circuits for J-turns and that a winner-take-all mechanism enables a specific behavior (J-turn vs. slow swim) to be selected (Fajardo et al., 2013). While early larvae approach *Paramecia* in an iterative fashion, juveniles strike from comparatively long distances (Figures 8, 13) and with sufficient precision that their trajectories accurately and quickly strike small targets (Figures 3, 5, 9 and 12).

Larval zebrafish have slow and fast motor systems which manifests as distinct “gaits” for the slow vs. fast/burst swims (Thorsen et al., 2004; Green and Hale, 2012). They depend upon red and white muscle (Buss and Drapeau, 2002) and distinct spinal oscillators or CPGs (Budick and O'Malley, 2000; Buchanan, 2001; Bhatt et al., 2007; McLean et al., 2008). The slow swims used in prey tracking are identical to those used in other larval behaviors (McElligott and O'Malley, 2005) and have been modeled in regards to CPG-frequency modulation (Hill et al., 2005; Kuo and Eliasmith, 2005; Knudsen et al., 2006). The high TBFs seen in early larval strikes are within the range of larval burst swim TBFs (Budick and O'Malley, 2000; Müller and van Leeuwen, 2004; Bhatt et al., 2007; McLean et al., 2008) but gradually decline over the next several weeks (Figure 6C). While the descending commands for predatory vs. other behaviors must differ, parsimony suggests conserved use of slow and fast spinal motor systems given the limited number of spinal interneuron classes seen anatomically (Bernhardt et al., 1990; Hale et al., 2001). These spinal networks could potentially be reconfigured to support the generation of a diverse variety of behaviors (Marder and Bucher, 2007; Bargmann, 2012).

The kinematic diversity of homing strikes (Figure 9) suggests further organizational capability, namely flexible composition of underlying neural commands in relation to the prey's distance and direction. For example, *Strike #1* is accomplished by a single slow bend (turn), reflecting an asymmetric motor command. *Strike #2* begins with a similarly low-angular velocity turn, but it then transitions into a low TBF forward swim bout, whose controlling neurons remain unknown (but see Green and Hale, 2012). In *Strike #2* slow swimming ceases after ~ five cycles, just before the moment of capture. The mechanism for halting in zebrafish is unknown, but based on *Xenopus* studies might reflect an explicit “halting” command (Perrins et al., 2002), or the run-down of spinal CPG activity after an initial, excitatory forward swim command (Dale, 1998). Inertia allows the juvenile in *Strike #2* to coast through the *Artemia*'*s* location, with suction jaw movements executed about 1080 ms after onset of the episode.

Juvenile *Strike #3* has some overlap with *Strike #2* in utilizing a command system that produces caudally-propagating bends, but at no time is a conventional slow-swimming pattern seen. *Strike #3* contains only 3 bends in total (vs. the 11 bends in *Strike #2*, and just one bend in *Strike #1)*. Of particular note is the bent posture of the trunk which is held between ~50 and 250 ms post-initiation of the episode. The kinematics suggests that sustained (i.e., non-oscillating) muscle contraction occurs concurrently with propulsive bends that propagate into the caudal trunk. This diversity of homing strikes rules out the use of rote, generic sensorimotor transformations and instead suggests that specific types of commands and command sequences are selected as a motor package based on the exigencies of each predatory opportunity. Such a process might depend on maturing forebrain structures, including an ancestral basal ganglia found in fishes (Medina and Reiner, 1995; Mueller et al., 2008; Ganz et al., 2012).

### Motor learning vs. innate knowledge

The initial prey-tracking and strike maneuvers are not “learned” behaviors, since they are performed with precision on the very first predation attempts of early larvae (Borla et al., 2002; McElligott and O'Malley, 2005; McClenahan et al., 2012), in agreement with observations of other larval fishes (Drost, 1987; Job and Bellwood, 1996). These sensorimotor programs thus constitute a kind of “innate knowledge” of predatory opportunities in that they were learned over evolutionary time, stored in the developmental programs of the zebrafish genome and are expressed through development (Adami et al., 2000; McNamara et al., 2006), aided presumably by ongoing sensory feedback that influences general motor and visual system development. The extent to which transitional behaviors and subsequent homing strikes might be innate is uncertain. We did not observe repetitive efforts to strike at a particular location in the arena. Had such repetition existed, larvae could in principle have utilized error signals and cerebellar learning mechanisms to improve performance, but the paucity of errors in conjunction with variable strike patterns, does not fit with conventional error-correction learning algorithms (Portugues and Engert, 2011; Ahrens et al., 2012). While zebrafish larvae exhibit cerebellar function and associative learning (Aizenberg and Schuman, 2011; Valente et al., 2012), the vanishing number of misses with age, along with good success at a young age (roughly 90% hits at 24 dpf; Figure 12), further suggests that growing zebrafish have either extremely fast sensorimotor learning algorithms or employ a series of innate motor programs that appear sequentially across ontogeny. A hybrid mechanism, whereby innate capabilities amplify larval learning skills, is perhaps most parsimonious.

### Conflict of interest statement

The authors declare that the research was conducted in the absence of any commercial or financial relationships that could be construed as a potential conflict of interest.
